# Serial ultra‐deep sequencing of circulating tumor DNA reveals the clonal evolution in non‐small cell lung cancer patients treated with anti‐PD1 immunotherapy

**DOI:** 10.1002/cam4.2632

**Published:** 2019-11-06

**Authors:** Li Li, Yubo Wang, Weiwei Shi, Mengxiao Zhu, Zhulin Liu, Nuo Luo, Yanwu Zeng, Yong He

**Affiliations:** ^1^ Department of Respiratory Disease Daping Hospital Army Medical University Chongqing China; ^2^ OrigiMed Co. Ltd Shanghai China

**Keywords:** ctDNA, distant metastasis, lung cancer, maximum somatic allele frequency, pembrolizumab

## Abstract

**Background:**

Immune‐therapy with anti‐PD1 inhibitors, such as pembrolizumab, is revolutionizing the treatment of non‐small cell lung cancers (NSCLC). However, identifying patients for the potential therapeutic response and predicting therapy resistance and early relapse remains a challenge.

**Methods:**

Between 2016 and 2018, 60 patients were treated with pembrolizumab, among who 12 NSCLC patients had both baseline (before treatment) and serial (on treatment) periodical circulating tumor DNA (ctDNA) samples. Those samples were sequenced on a 329 pan cancer‐related gene panel. Analyses of tumor burden, blood tumor mutational burden (bTMB), maximum somatic allele frequency (MSAF), and tumor clonal structure were performed in association with clinical response. Candidate resistance mutations involved in relapse and metastases were further investigated.

**Results:**

ctDNA was detected and mutational profiling was performed for each patient. Those with a high baseline bTMB level showed significantly improved progression‐free survival (PFS) after pembrolizumab treatment. Tumor burden and therapeutic response significantly correlated with the MSAF instead of the bTMB. Clone analysis detected tumor progression about 2‐4 months ahead of computed tomography (CT) scan. One mutation in gene *PTCH1* (Protein patched homolog 1) and two acquired anti‐PD1 candidate resistance mutations of gene *B2M* (β2 microglobulin) were identified in association with distant metastasis. The evolutionary tree of a representative patient was also described.

**Conclusion:**

This pilot study showed that MSAF could be another good indicator of therapeutic response, and clonal analysis could be clinically useful in monitoring clonal dynamics and detecting remote metastasis and early relapse.

## Graphical abstract

MSAF as a good indicator of therapeutic response for immunotherapy in lung cancer; ctDNA sequencing for monitoring clonal dynamics, and detecting remote metastasis and early relapse

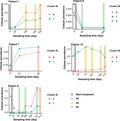

## 1 INTRODUCTION

Lung cancer is the leading cause of cancer‐related deaths worldwide, of which non‐small cell lung cancer (NSCLC) comprises more than 80%. Recently, immune checkpoint inhibitor therapy changed the treatment of NSCLC. One main drawback of it is the lack of appropriate biomarkers to track treatment response. Current monitoring approaches, including CT scan, have some limitations such as pseudo‐progression and pseudo‐response problems.^1^, ^2^, ^3^, ^4^

Circulating tumor DNA (ctDNA), which is fragmented DNA releasing from tumor cells through apoptosis or necrosis, is an emerging noninvasive biomarker. Several groups have proven that ctDNA can be detected in the plasma across a wide range of cancer types.^5^, ^6^, ^7^ For example, the detection rate is ~50% in early‐stage tumors and 80%‐100% in metastatic disease in NSCLC.^6^, ^7^ ctDNA can be used to track tumor burden in response to immunotherapy. It has been demonstrated to have higher sensitivity than a CT scan in several studies.^5^, ^8^, ^9^ However, most of these studies require a baseline tissue biopsy to determine somatic mutations and follow‐up with low‐throughput PCR methods such as droplet digital PCR (ddPCR). It not only imposes a challenge for acquiring tissue from late‐stage patients but also limits the use of detecting mutations in distant metastasis or acquired resistance for immunotherapy. For example, mutations' occurring in the antigen presentation machinery is a known acquired resistance mechanism.^10^ Here we hypothesized that ctDNA mutation profiling on pan cancer‐related gene panel sequencing could simultaneously monitor treatment response and predict candidate resistance mutations and clones, including those from distant metastasis.

In this study, we collected multiple baseline and on‐treatment ctDNA samples in NSCLC patients treated with a PD1 (Programmed Death‐1) inhibitor, pembrolizumab. We profiled the ctDNA by an ultra‐deep sequencing for a pan cancer‐related gene panel. By analysis of the ctDNA profile for each patient, we studied the correlation between ctDNA and treatment efficacy and identified potential anti‐PD1 candidate resistance mutations and private subclonal metastasis mutations.

## 2 MATERIALS AND METHODS

### 2.1 Patient enrollment, treatment and clinical outcomes evaluation

This study was approved by the ethical committee of Army Medical University, and written consent was received from all patients. This is a real‐world study and the aim of the study is to observe whether ctDNA analysis can monitor PD‐L1 inhibitor efficacy. Patients were treated with pembrolizumab either as first‐line or second‐line therapy, with or without combined paclitaxel and cisplatin chemotherapy. The dose of pembrolizumab was 200 mg every three weeks. The patient inclusion criteria are: (a) Advanced non‐small cell lung cancer (stage IIIB or stage IV); (b) Patients were treated with pembrolizumab either as first‐line or second‐line therapy, with or without combined paclitaxel and cisplatin chemotherapy; (c) Written consent was received; (d) Blood samples were collected from patients. The patient exclusion criteria are: (a) Patients without radiological assessments; (b) Patients without blood samples; (c) Patients received pembrolizumab as neo‐adjuvant therapy.

Two patients were PD‐L1 positive detected by immunohistochemistry staining (Figure S1) with over 1% PD‐L1 positive cells in the tumor tissues. Of the other 10 PD‐L1 negative patients (Table 1), 3 received second‐line IO (patient 2, 9, 12) and 7 received first‐line IO (patient 1, 3, 4, 5, 8, 10, 11). Of the 7 patients, 2 received combined pembrolizumab + chemotherapy and 5 received pembrolizumab alone. Of the 5 patients, 3 were PD‐L1 negative and 2 patients had not enough sample for PD‐L1 testing. For those 5 patients, the standard of care should be chemotherapy. However, they refused chemotherapy and requested immunotherapy as monotherapy.

**Table 1:** Clinicopathologic characteristics of patients (n = 12)

Patient ID	Age	Gender	Smoking index	Staging	Diameter sum (cm)	Histology	Treatment	Combined chemotherapy	Best response	Second‐ line IO	PD‐L1
1	50	male	400	IV	5.2	Squamous cell carcinoma	pembrolizumab	No	SD	No	−
2	59	male	1500	IV	4.2	Squamous cell carcinoma	pembrolizumab	No	SD	Yes	−
3	61	male	800	IV	10.9	Squamous cell carcinoma	pembrolizumab	Yes	PR	No	−
4	68	male	600	III	5	Squamous cell carcinoma	pembrolizumab	Yes	SD	No	−
5	72	male	800	IV	4.7	Squamous cell carcinoma	pembrolizumab	No	PR	No	−
6	54	male	600	IV	5.1	Squamous cell carcinoma	pembrolizumab	No	PR	Yes	+
7	66	male	0	IV	28.7	Squamous cell carcinoma	pembrolizumab	No	PD	No	+
8	63	female	0	IV	34.1	Squamous cell carcinoma	pembrolizumab	No	PR	No	NA
9	69	male	450	IV	8	Squamous cell carcinoma	pembrolizumab	No	PR	Yes	−
10	57	male	800	IV	8.7	Adenocarcinoma	pembrolizumab	No	SD	No	−
11	64	female	0	IV	3.4	Squamous cell carcinoma	pembrolizumab	No	SD	No	NA
12	60	male	800	IV	6.4	Adenocarcinoma	pembrolizumab	No	SD	Yes	NA

Note

The smoking index was defined as cigarettes per day multiplied by years smoked; Diameter is the longest diameter of the target lesion. IO, immuno‐oncology.

**Figure 1:**
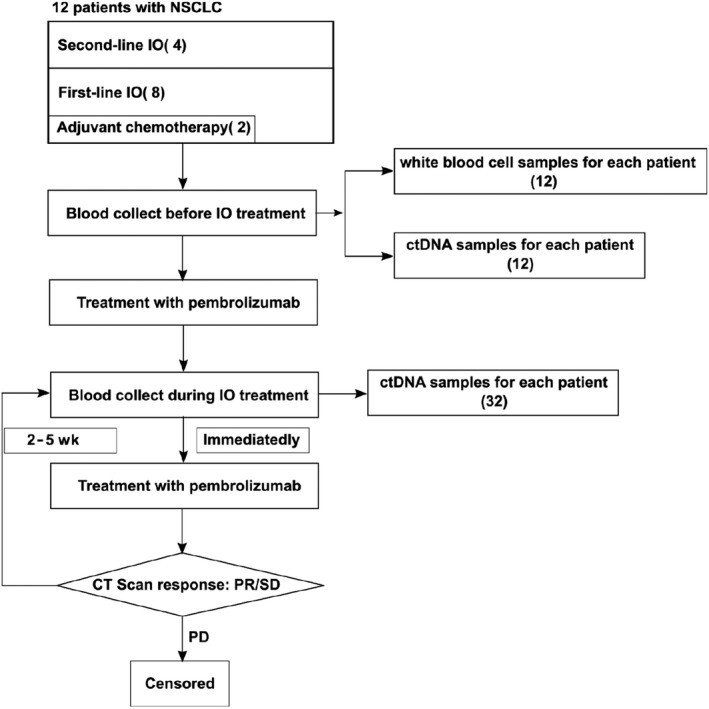
Patient enrollment for treatment and sample collection

CT scans were conducted periodically (Figure 1). The clinical outcomes were described as partial response (PR), stable disease (SD) and progressive disease (PD) according to iRECIST (Immune‐Modified Response Evaluation Criteria In Solid Tumors),^11^ a modification from the RECIST system.^12^ Progression‐free survival (PFS) was defined as the interval between the initial time of treatment administration and the time of PD or that of the last follow‐up. The smoking index was defined as cigarettes per day multiplied by years smoked.

### 2.2 Immunohistochemistry

The formalin‐fixed paraffin‐embedded (FFPE) tissue from lung tumor was prepared for immunohistochemistry staining of PD‐L1. The glass slides were dewaxed by heating in a 60°C oven for one hour, and washing (3×) for 5 minutes with xylene. Then they were rehydrated (4x) with alcohol, each time in a different concentration (100%, 95%, 85%, and 75%). Antigen retrieval was applied in a pressure cooker for 4 min and then a cold water bath for 10 minutes. The slides were blocked with 10% goat serum for 30 minutes, and stained with primary mouse anti‐human PD‐L1 antibody (Abcam, clone 28‐8, #ab205921, diluted 1:300) for 50 minutes, followed by secondary antibody biotinylated with HRP (horseradish peroxidase) for 25 minutes (DAKO, K8002 kit). Next, the slides were stained with diaminobenzidine for five min and enhanced for five min (DAKO, K8002 kit). They were then restained with hematoxylin for 1.5 minutes, washed with alcohol of gradient concentrations (75%, 85%, and 95%) and dehydrated with xylene for five min. Samples with a positive cell percentage greater than 1% were defined as PD‐L1 positive.

### 2.3 ctDNA and white blood sample collection and library preparation

From each patient, blood samples (8‐10 mL) were collected about once per month. In order to separate cell‐free DNA (cfDNA), the blood samples were centrifuged (2× at 1600 *g* for 10 minutes and 15 minutes, respectively) and the supernatant was separated. cfDNA was extracted from 5ml of plasma with QIAamp^®^ Circulating Nucleic Acid Kit (Qiagen, Venlo, Netherlands, #55114). If the volume was less than 5 mL, PBS (phosphate buffered saline) would be added to make all samples of equal volume. cfDNA concentration was determined with the Invitrogen Qubit^®^ DNA HS Assay Kit (#Q32854), excluding single strand DNA and protein contamination. Samples with at least 50 ng of double‐stranded DNA extraction were used for library construction. Molecular identifiers (MIDs) were added to the DNA segment ends for cfDNA libraries in order to reduce the false discovering rate (FDR). Additional sample indices (barcodes) for multiplex sequencing were added for both types of libraries.

### 2.4 Ultra‐deep sequencing and variants calling

The ctDNA libraries were enriched with a pan‐cancer ctDNA panel (Qiyuan^®^, Origimed Co., Ltd) consisting of 329 genes (Table S1) and targeting 637k base pairs. For each patient, the library of white blood samples was also enriched with the whole‐exome panel (SureSelect^®^ Human All Exon V6, Agilent). Both types of libraries were sequenced on Illumina Novaseq 6000 (Illumina) for 151bp read length from both ends. For the ctDNA panel and whole‐exome sequencing, the clean reads coverage was about 3000× and >100× on average for ctDNA and white blood libraries, respectively.

Raw reads from ctDNA and whole‐exome sequencing were trimmed for adaptors by cutadapt (version 1.18).^13^ An additional deduplication of MID‐labeled reads was applied on ctDNA samples with an in‐house pipeline. High‐quality reads were mapped to the UCSC hg19 reference sequences with BWA MEM (version 0.7.9a).^14^ Base quality was recalibrated by the BaseRecalibrator tool from GATK (version 3.8).^15^ Variants from ctDNA were detected, using Mutect2 with tumor only mode.^16^ Germline variants from the white blood samples were identified using Varscan (version 2.3.9)^17^ with parameters "mpileup2cns ‐‐strand‐filter 0 ‐‐min‐coverage 1 ‐‐min‐reads2 3 ‐‐min‐avg‐qual 0 ‐‐min‐var‐freq 0.0001 ‐‐p‐value 1".

For each cfDNA sample, the germline filtering steps were as follows: 1. Common germline variants having variant allele frequency (VAF) <0.1% were filtered according to the databases of ESP6500,^18^ 1000 Genomes,^19^ gnomAD,^20^ and ExAC^21^; 2. Patient‐specific germline variants were further filtered if found with more than two reads in the white blood samples from the same patient; 3. If a candidate somatic variant occurred with VAF >20% across all time points for a patient, it was likely to be a private germline variant and was also filtered; 4. Mutations with maximum VAF change >2% among all time points for a patient were manually checked with IGV.^22^ Somatic variants that had not been filtered were further annotated by ANNOVAR (2017/07/17)^23^ with RefSeq (version 2017/06/01).

### 2.5 Bioinformatics analysis

The maximum somatic allele frequency of mutations (MSAF) was computed for each sample from reportable genomic alterations (GAs), variants of unknown significance and synonymous mutations^24^ by measuring the maximum VAF of all passing somatic variants. Clonal deconvolution analysis was conducted with Python package "PyClone".^25^ Copy number information at each mutation location was computed by CNVKit.^26^ The copy number was assigned 1 for logR values below −0.25, 3 for logR values above 0.25 and 2 for logR values in between −0.25 and 0.25.^27^

### 2.6 Statistical analysis

All statistical tests were applied with R software. Mann‐Whitney *U* test was used to test the association between MSAF and clinical outcome. Survival analysis and Cox proportional hazard analysis was performed using package "survival" and plotted with package "survminer".^28^ The log‐rank test was utilized to compare PFS differences.

## 3 RESULTS

### 3.1 Clinicopathologic characteristics and sample sequencing

The patient enrollment was described in the method section. In this study, 12 NSCLC patients who had both baseline (before treatment) and serial (on treatment) periodical circulating tumor DNA (ctDNA) samples were studied. Clinicopathologic characteristics are summarized in Table 1. All patients were staged according to the 8th edition of the tumor‐node‐metastasis (TNM) classification system for lung cancer from the American Joint Committee. Eleven patients were at Stage IV, and one was at Stage III. Nine patients were heavy smokers with a smoking index of at least 400, and the other three patients were nonsmokers. Two patients were PD‐L1 positive detected by immunohistochemistry staining (Figure S1) with over 1% PD‐L1 positive cells in the tumor tissues. Of the other 10 PD‐L1 negative patients (Table 1), 3 received second‐line IO (patient 2, 9, 12) and 7 received first‐line IO (patient 1, 3, 4, 5, 8, 10, 11). Five of the 12 patients (42%) had a PR response after the first treatment.

The sample collection procedure was shown in Figure 1. In addition to the baseline, peripheral blood was collected every 2‐5 weeks continuously. In total, 44 samples were prepared for ultra‐deep ctDNA panel and sequenced with average coverage depth about 3000× after deduplication. White blood cells from each patient were also collected, sequenced (average coverage depth >100×) and analyzed to exclude germline variants and clonal hematopoietic mutations.^29^

### 3.2 Monitoring tumor burden in NSCLC with ctDNA

**Figure 2:**
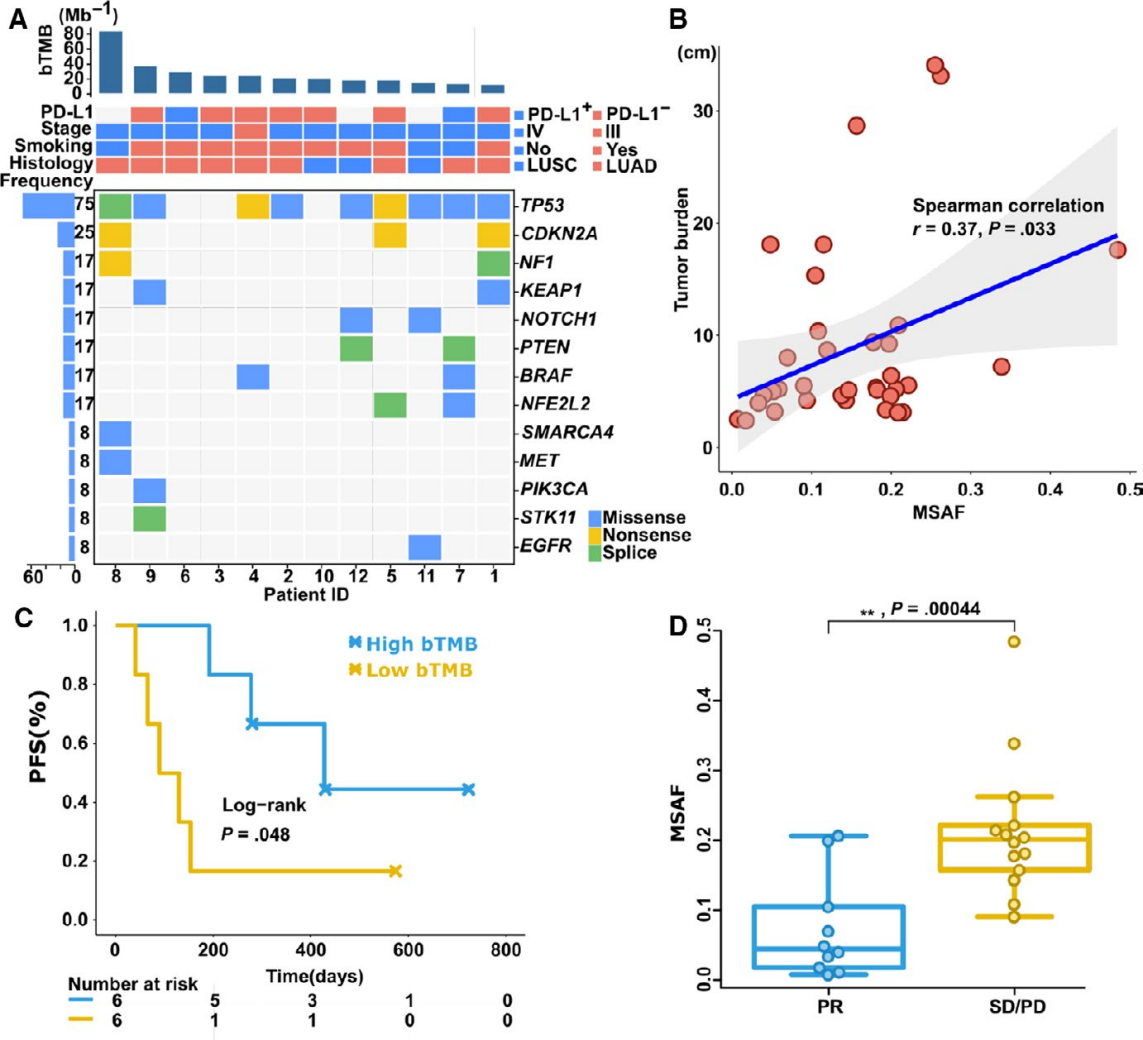
ctDNA could monitor tumor burden and predict progression‐free survival. A, The topmost panel shows the distribution of bTMB (blood tumor mutational burden). Twelve patients were arranged along the x‐axis in descending order of bTMB. The middle panel indicates the clinical information for each patient. The bottom panel shows 13 somatic mutations of lung cancer‐specific driver genes and the bottom left panel shows the frequency of each gene mutation. LUSC, squamous cell carcinoma; LUAD, lung adenocarcinoma. B, Scatter plot between MSAF and tumor burden. The Spearman's rank test showed a significant correlation (<0.05). C, Lower bTMB (<median = 21/Mb) had a better PFS. D, The mean MSAF (mMSAF) for each patient was significantly higher for the SD/PD outcome than PR outcome (Mann‐Whitney *U* test)

Twelve pretreatment ctDNA samples were sequenced on a 329 cancer gene panel (Table S1). Raw reads were then trimmed of barcodes, deduplicated by MIDs and mapped onto hg19 (UCSC hg19/GRCh37). Single‐nucleotide variants were detected with mutect2.^16^ Germline variants were filtered by whole‐exome sequencing of the matched white blood cells and with a public population database. Figure 2A shows the mutations of high‐confident NSCLC driver genes at the baseline of these 12 patients, reported by TCGA (The Cancer Genome Atlas).^30^, ^31^ Among these driver gene mutations, *TP53* and *CDKN2A* were the most frequently mutated with frequencies of about 75% and 25%, respectively. No apparent driver gene mutation was found in patient 3, 6 or 10. Only *TP53* showed significant difference between PR and SD group (*P*‐value = .045, single‐tail Wilcox test).

Previous studies showed ctDNA fraction was associated with clinical response.^32^, ^33^ As a measure of ctDNA fraction, MSAF was calculated for each sample. Here we attempted to find whether or not ctDNA fraction was associated with tumor burden. To remove the high ctDNA frequency possibly caused by aneuploidy, Gandara et al set a maximum threshold of 20% for each mutation,^34^ but this may be too restricted for our cases. Instead, we took into account the VAFs across all of the samples by each patient. Only mutations with VAF >20% across all patient samples were removed. Thus, for the total 44 samples, the resulting MSAF ranged from 0.0075 to 0.48. A significant linear correlation was observed between tumor burden and MSAF (Spearman correlation coefficient = 0.37, *P*‐value = .033) (Figure 2B).

### 3.3 Predicting clinical outcome with ctDNA

In order to explore the potential of ctDNA as a predictor of PFS, we first tested the baseline ctDNA assay‐based bTMB as a response predictor. Kaplan‐Meier analysis showed that the baseline bTMB significantly correlated with the PFS of our patients (Log‐rank test, *P*‐value = .048) (Figure 2C). Higher baseline bTMB (>median = 21/MB) showed a significantly better PFS. Next, we tested bTMB and MSAF as a response predictor for clinical outcome. The responses were assessed periodically in each patient by CT scan. Accordingly, 32 plasma samples were classified into two groups: PR and SD/PD. While compared by the Mann‐Whitney U test, the bTMB in the PR group was not significantly different from that in the SD/PD group (Figure S2, Mann‐Whitney *U* test, *P*‐value = .29). However, the MSAF of samples in the SD/PD group was significantly higher than that in the PR group (Figure 2D, Mann‐Whitney *U* test, *P*‐value = .00044).

### 3.4 ctDNA identified early resistance and remote mutations

**Figure 3:**
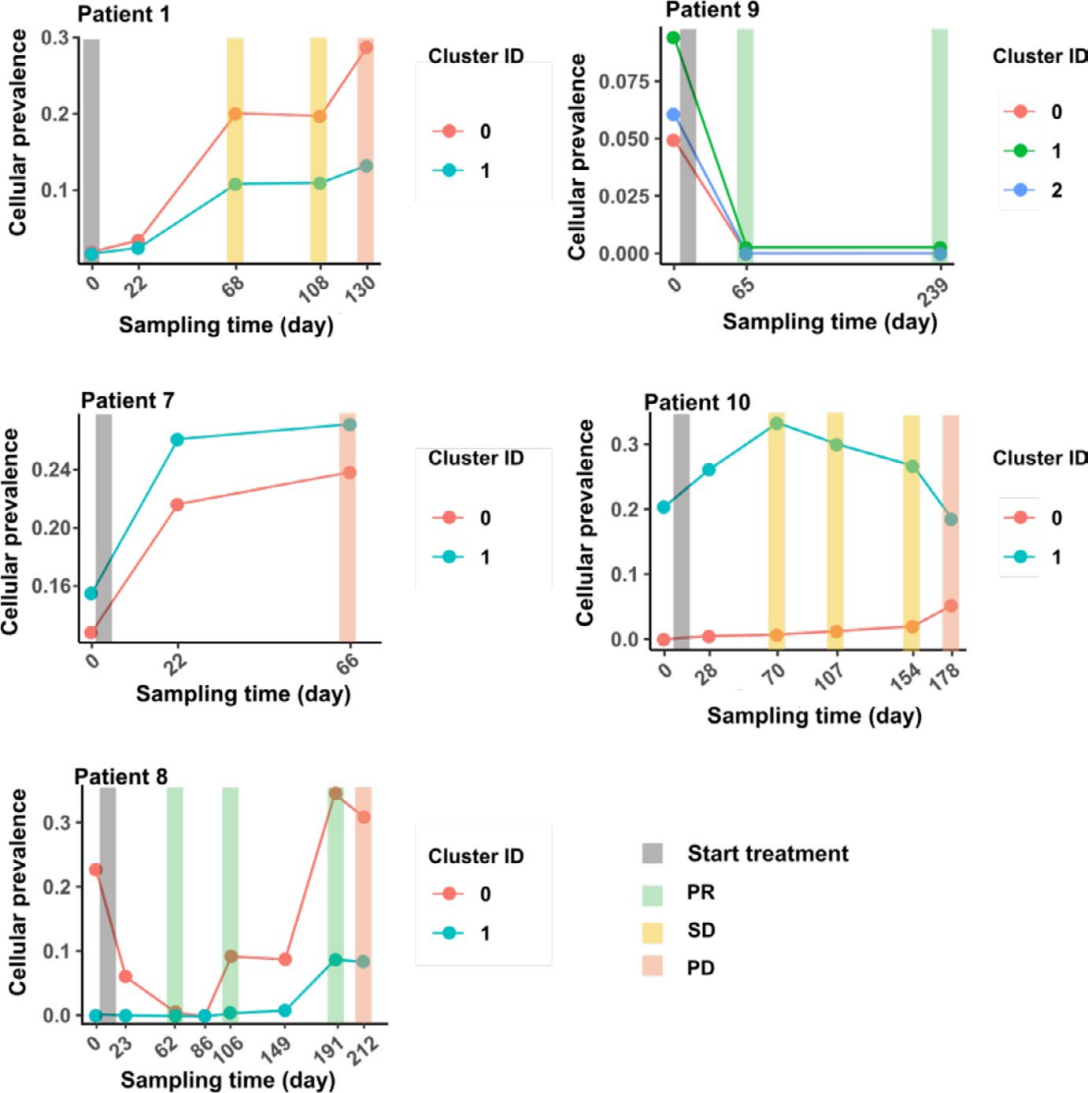
The cellular frequency of mutation clusters in five patients. The cellular frequency of mutation clusters from PyClone analysis in five patients. The x‐axis shows the sampling time (days) and the y‐axis shows the cellular frequency. The colored bar along the x‐axis indicates the treatment response

PyClone^25^ was applied to deconvolute mutations into different clusters and predict the clonal fraction of each cluster across multiple time points. To ensure a reliable parameter estimate, only patients with at least three time points were selected. Five patients had passed the filtering criteria (Figure 3). Of those five patients, one patient achieved PR and four had PD at the end of the treatment. In all five patients, ctDNA was able to detect the clones 2‐4 months ahead of a CT scan‐confirmed relapse.

**Figure 4:**
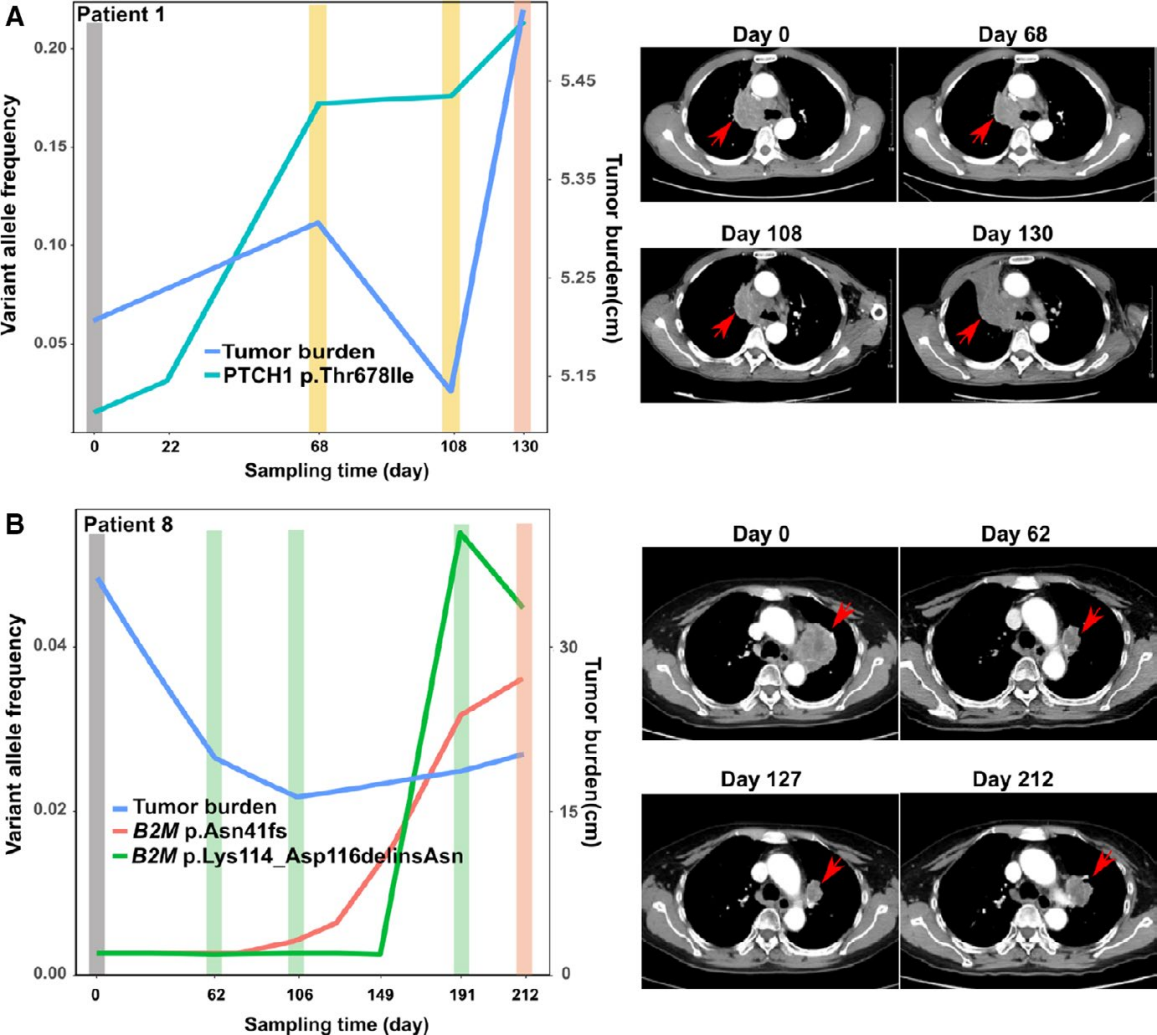
Mutations were associated with immunotherapy resistance. Tumor burden and VAF are shown on left and CT images are shown on the right for patient 1 and 8, respectively. The red arrows indicated the tumor position. A and B, The *PTCH1* mutation (p.Thr678Ile) in patient 1 is shown in (A). The two mutations in *B2M* (p.Asn41fs and p.Lys114_Asp116delinsAsn) are shown in (B)

Specifically, ctDNA‐based clonal analysis results from patient 1 and patient 8 were compared with CT scan images (Figure 4). In patient 1, *PTCH1* (p.Thr678Ile) with the rising VAF reflected the relapse more than three months ahead of the CT scan (Figure 4A, Figure S3A). Another interesting case was patient 8 who first achieved PR and then had confirmed PD characterized by increased tumor size across primary, liver and paraspinal metastasis sites (Figure S4B). This patient specifically had a *B2M* mutation (p.Asn41fs), which was detected after three months of treatment. It was nearly 4 months ahead of the relapse detected by a CT scan. Furthermore, another acquired *B2M* mutation (p.Lys114_Asp116delinsAsn) was also identified one month ahead of the CT scan (Figure 4B). *PTCH1* and *B2M* mutation was not found in the other patients.

Moreover, we studied the association between mutations and specific metastasis sites. For patient 1, the primary tumor reduced between day 0 and 108. However, a new lesion in the scapula was detected on day 68 (Figure S3A). During this period, the frequency of the *PTCH1* mutation increased rapidly while the primary lesion decreased (Figure 4A), which suggested it may originate from scapula metastasis rather than the primary lesion (Figure S3A). For patient 8, on day 127, *B2M* mutation (p.Asn41fs) had increased and, at the same time, the paraspinal metastasis progressed (Figure S3B) while the lung and liver tumor lesion decreased. The lung and liver tumor lesion grew between day 127 and day 212, which was consistent with the appearance of *B2M* mutation (p.Lys114_Asp116delinsAsn) between day 149 and 191. Taken together, it can conclude that *B2M* mutation (p.Asn41fs) should be associated with paraspinal metastasis, while *B2M* mutation (p.Lys114_Asp116delinsAsn) should be associated with the lung and liver tumor lesion.

### 3.5 Reconstruction of clonal evolutionary history

**Figure 5:**
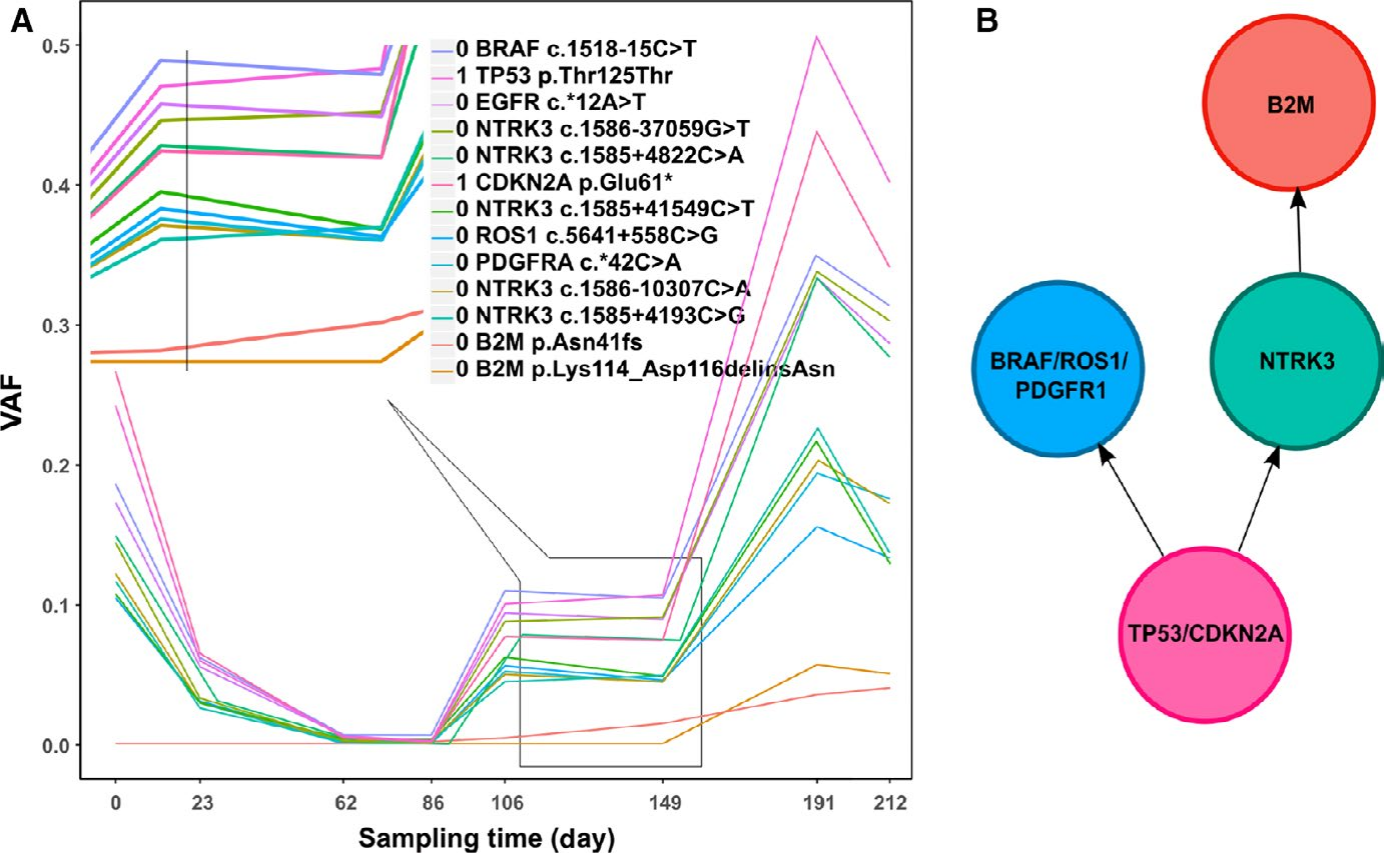
The evolutionary trajectory of sub‐clones within the ctDNA samples of patient 8. A, VAF vs. the sampling time for individual mutations. The mutations were annotated with cluster id and mutation name. The left top corner of the plot shows a zoomed‐in trend change between 106 and 149 days. B, The inferred evolutionary tree was showed with each circle indicating a mutation cluster

The patient 8 had samples at multiple time points and rich mutations, which provides a good example for studying tumor evolution. We constructed the tumor evolutionary tree based on the PyClone results and manual clustering of the VAF trend along the timeline. Firstly, the VAF of all mutations was plotted along the timeline (Figure 5A). The PyClone analysis gives two clusters, cluster 0 and cluster 1. The cluster ids were indicated in front of the gene names in Figure 5A. Cluster 0 (*TP53/CDKN2A*) should be the antecedent cluster which had the highest VAF. It was further divided into subgroups manually according to the frequency trend of the mutations. Between day 106 and 149, the four mutations (*TP53/NTRK3* c.1585+4193C>G/B2M) had an ascending trend while the other three mutations (BRAF/ROS1/PDGFR1) had a descending trend. For the former sub‐cluster (*TP53/NTRK3* c.1585+4193C>G/B2M), since *B2M* obviously appeared later than the other two mutations, the logical evolutionary trajectory for the *B2M* was *TP53/CDKN2A* to *NTRK3* to *B2M*. For the latter sub‐cluster (BRAF/ROS1/PDGFR1), they should be on the diverted evolutionary path from (*NTRK3* c.1585+4193C>G/B2M). The evolutionary tree was concluded in Figure 5B.

## 4 DISCUSSION

Immune checkpoint inhibitors, such as pembrolizumab, have shown significant clinical benefit in late‐stage NSCLC patients. However, it still remains a challenge in clinical management to predict treatment response and monitor disease progression. Traditional methods such as CT scans are often delayed to reflect disease progression. With superior performance compared to CT scans, ctDNA profiling, as used in this study, also has unique advantages over previous study designs. For example, there have been many studies related to ctDNA monitoring that usually sequence the tissue on a comprehensive assay and then monitor those mutations through ddPCR in ctDNA from different time points.^8^, ^35^, ^36^, ^37^ These approaches have the advantage of low cost and low input requirement on plasma for treatment monitoring; however, they are incapable of detecting subclonal or acquired candidate resistance mutations due to the limited probes and tissue availability. Few studies have sequenced ctDNA on a comprehensive assay to detect de novo mutations before and during treatment. Though whole‐exome sequencing can be used to track treatment process, it is too expensive to achieve enough coverage depth, leading to a low mutation detection rate and accuracy. Using a cancer‐related gene panel, as in our study, can better balance the detection sensitivity and cost, and maximize the application value. Besides, the tissue availability for late stage patients is a critical problem as in our study, where ctDNA panel sequencing is especially useful.

In this pilot cohort, we have found that baseline bTMB can predict patient response to pembrolizumab. Similar results for anti‐PD1/anti‐PD‐L1 treatment have also been found in recent studies.^34^, ^38^ Considering that tTMB (tumor mutational burden) is a good measure of overall neo‐antigen load, along with the positive correlation between tTMB and bTMB^34^ and the easy availability of blood biopsies, it is assumed that baseline bTMB would be a better outcome predictor than tTMB in the future clinical practice. Different from the baseline bTMB, MSAF is a good indicator of tumor burden during treatment. During treatment, MSAF showed a significant association with tumor burden and treatment outcome. A previous work by Demuth, Winther‐Larsen *et al* showed an insignificant association between ctDNA concentration and tumor burden,^8^ which might be due to different platforms (ddPCR vs. ctDNA panel) and measurements.

Subclones are considered responsible for drug resistance and disease progression.^39^ High‐quality VAF obtained from ultra‐deep panel sequencing can further be used in the clonal analysis, which can find clones responsible for early relapse and distant metastasis ahead of a CT scan. For example, in our study, a *PTCH1* mutation, p.Thr678Ile, was found four months ahead of a CT scan in patient 1. Loss‐of‐function of *PTCH1*, an inhibitor of smoothened (*SMO*) oncogenes, can cause nevoid basal cell carcinoma syndrome.^40^ Patients with relapse caused by such a mutation were previously treated with vismodegib, a small molecular inhibitor of SMO protein.^40^ The frequency of p.Thr678Ile gradually increased during treatment while the tumor burden showed a short‐term drop and then a quick increase. Evidence showed that aberrant sonic hedgehog pathway activation is associated with poor treatment outcomes, but it is still unclear whether the *PTCH1* mutation brought about the resistant response. Besides, we have also identified two acquired mutations in the *B2M* gene during treatment for patient 8. *B2M*, a component of MHC (major histocompatibility complex) Class‐I molecules, is important for antigen presentation in the adaptive immune system and is frequently the source of immunotherapy resistance.^10^ Recurrent inactivation of *B2M* has been discovered in lung cancer patient‐derived xenografts (PDX).^41^ The impaired HLA‐I complex by *B2M* could impair the response to anti‐PD‐1 therapy. In contrast to previous findings, *B2M* mutations might be responsible for the acquired resistance.

Along with CT scans, ctDNA panel sequencing can further associate the mutations with distant metastasis. For example, the mutation in *PTCH1* appeared to be associated with scapula metastasis in patient 1, whereas the other two *B2M* mutations, p.Asn41fs and p.Lys114_Asp116delinsAsn, seem to be associated with paraspinal metastasis and with lung and liver tumor lesions in patient 8, respectively. Though *PTCH1* and *B2M* gene mutation correlates with the tumor growth, but it needs more proof to check the underlying mechanism between *B2M* mutation and the resistant response.

Our two‐step evolutionary tree analysis illustrated how a clone with a *TP53* gene mutation positively evolved to escape from immune therapy in patient 8. The manual check of VAF was based on the simple rule: the antecedent cluster showed sum‐up pattern in its descendent clusters along the following time points. Thus, the descendent cluster must exhibit a smaller cellular prevalence than its antecedent cluster, which was called "crossing rule".^29^ Initially, the highest mutational gene, *TP53*, was mutated and the DNA repair function was impaired. Many mutations emerged including *CDKN2A*, which is related to cell proliferation. Tumor cells harboring these two mutations accumulated quickly along with many other mutations, including an important mutation of *NTRK3*. The mutations, *c.1585+4193C>G* in the intron of *NTRK3* and *p.Asn41fs* in the exon 2 of *B2M*, had a similar trend between day 86 and 149, suggesting that the latter mutation was likely within the former clones. After day 149, another novel mutation, *p.Lys114_Asp116delinsAsn*, also in the exon 2 of *B2M*, appeared within the clone with the *NTRK3 c.1585+4193C>G* mutation, accelerating disease progression. Presumably, the key event prior to relapse was the *NTRK3* mutation, which could be a target for anti‐evolution therapy.^35^ It must point out that the above inference is only based on a simple association analysis and more proof is needed.

To minimize contamination from germline variants and potential clonal hematopoietic mutations, we not only used the public database but also sequenced the exome of white blood cells to filter out them. Moreover, multiple samples from the same patient were combined to remove the extraordinarily high frequency alleles, avoiding using the maximum allele frequency of any one particular sample as a threshold. For PyClone analysis, such careful filtering of false positive mutations is critically important.

Limitations of our study included small sample size, which weakens the statistical significance of our conclusions. For example, clinical outcomes for high bTMB is better than low bTMB (Figure 2C); *TP53* showed a significant difference between PR and SD group; despite the high rate of negative PD‐L1, this study showed a higher objective response rate (42%) than keynote‐042 (27%‐39%),^42^ which could benefit from the first‐line or combined chemotherapy. All these observations need more samples to test.

In summary, this pilot cohort suggests that serial ultra‐deep sequencing of ctDNA on a comprehensive cancer panel can be an effective approach to track clonal evolution, which can assess tumor response in patients treated with anti‐PD1 therapy. Further study involving a larger cohort of NSCLC patients is needed to validate our findings.

## CONFLICT OF INTEREST

The authors declare no potential conflicts of interest.

## Data Availability

The data that support the findings of this study are available from the corresponding author upon reasonable request.
