# Supplementary material for: Serial ultra‐deep sequencing of circulating tumor DNA reveals the clonal evolution in non‐small cell lung cancer patients treated with anti‐PD1 immunotherapy
Source: Cancer Med. 2019 Nov 6;8(18):7669–78. doi: 10.1002/cam4.2632 (PMC6912064; doi:10.1002/cam4.2632)
Supplement: Supplementary file 1 [file CAM4-8-7669-s001.pptx]

## Slide 1
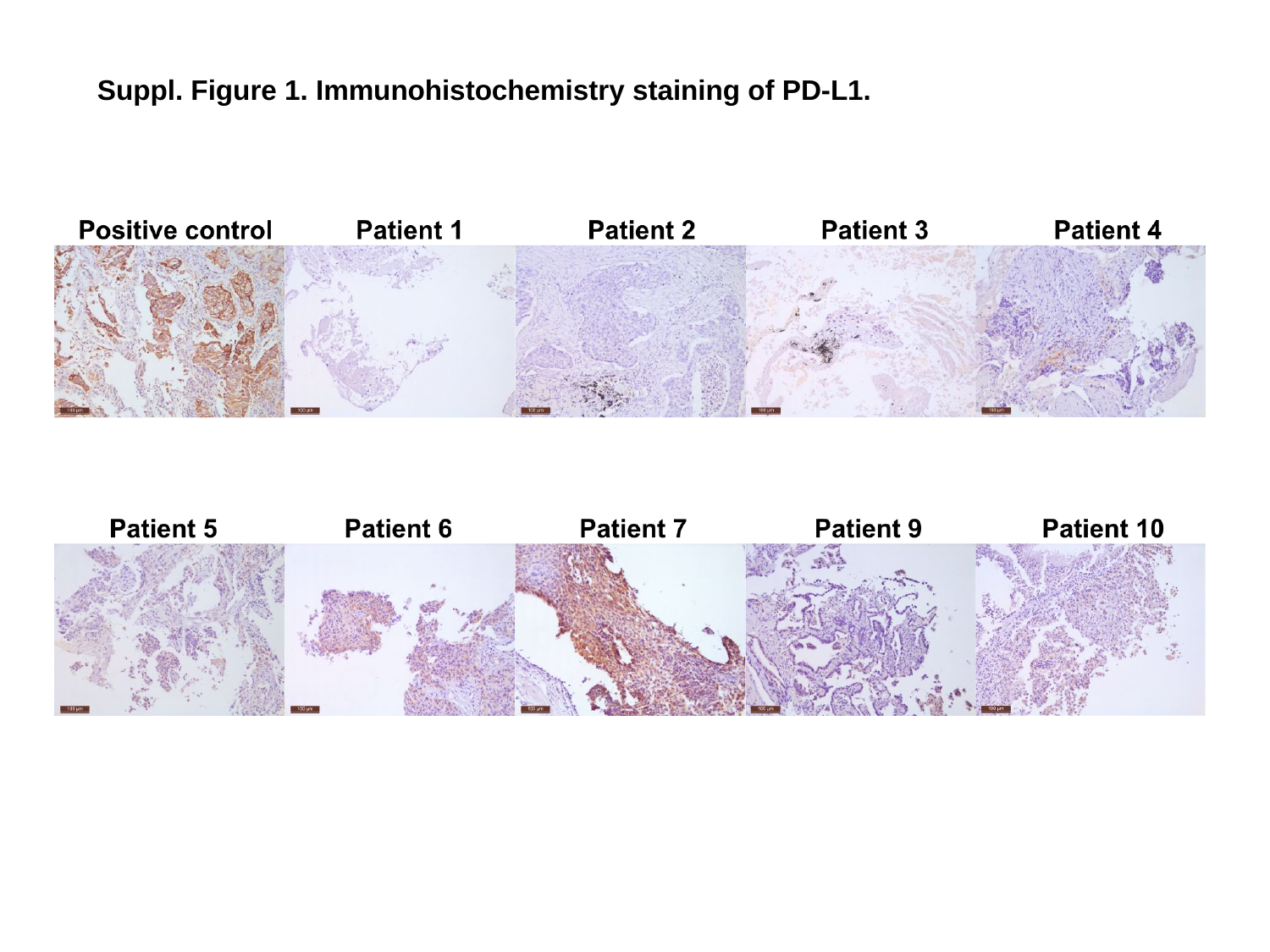

Suppl. Figure 1. Immunohistochemistry staining of PD-L1.

## Slide 2
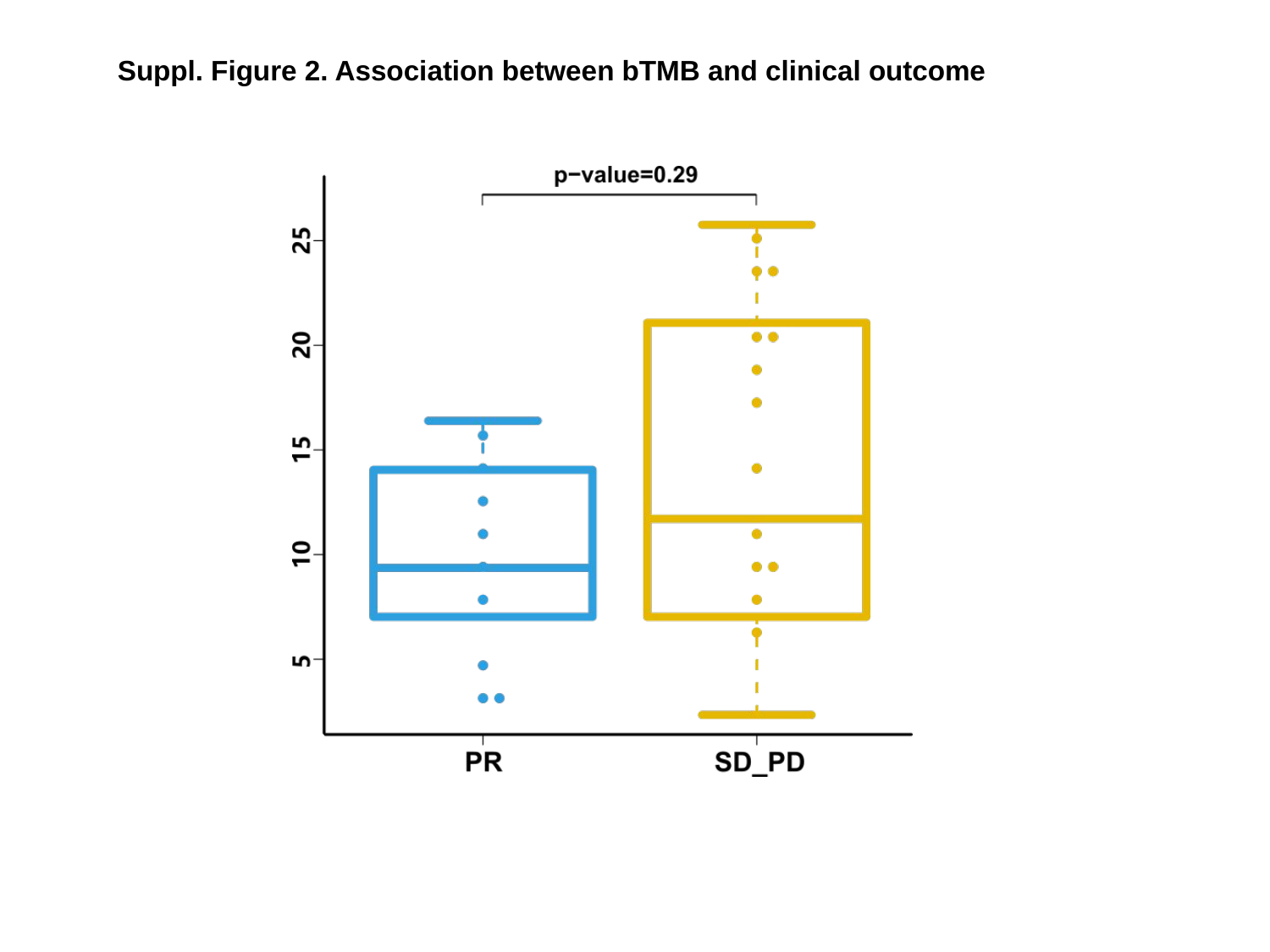

Suppl. Figure 2. Association between bTMB and clinical outcome

## Slide 3
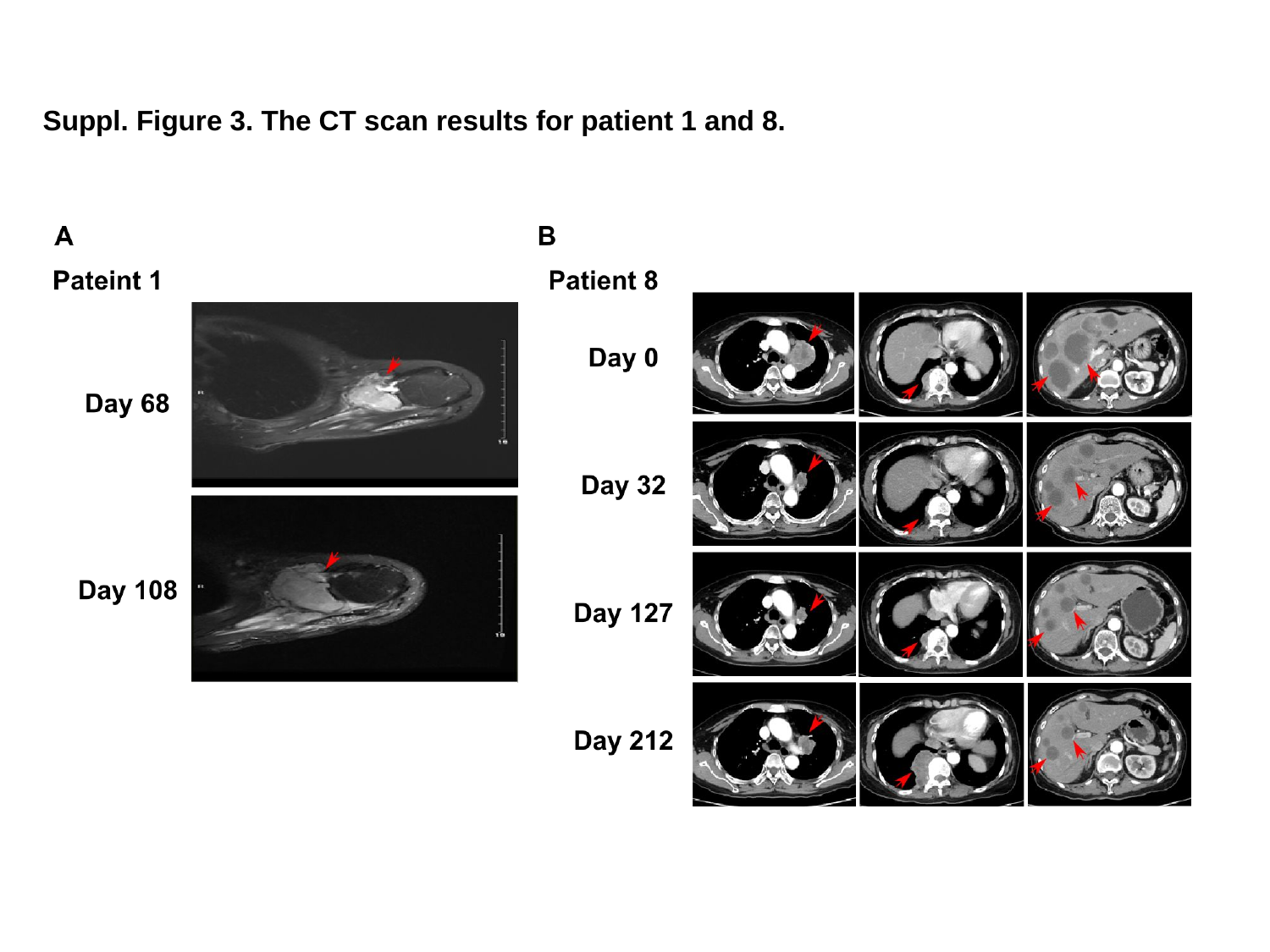

Suppl. Figure 3. The CT scan results for patient 1 and 8.

## Slide 4
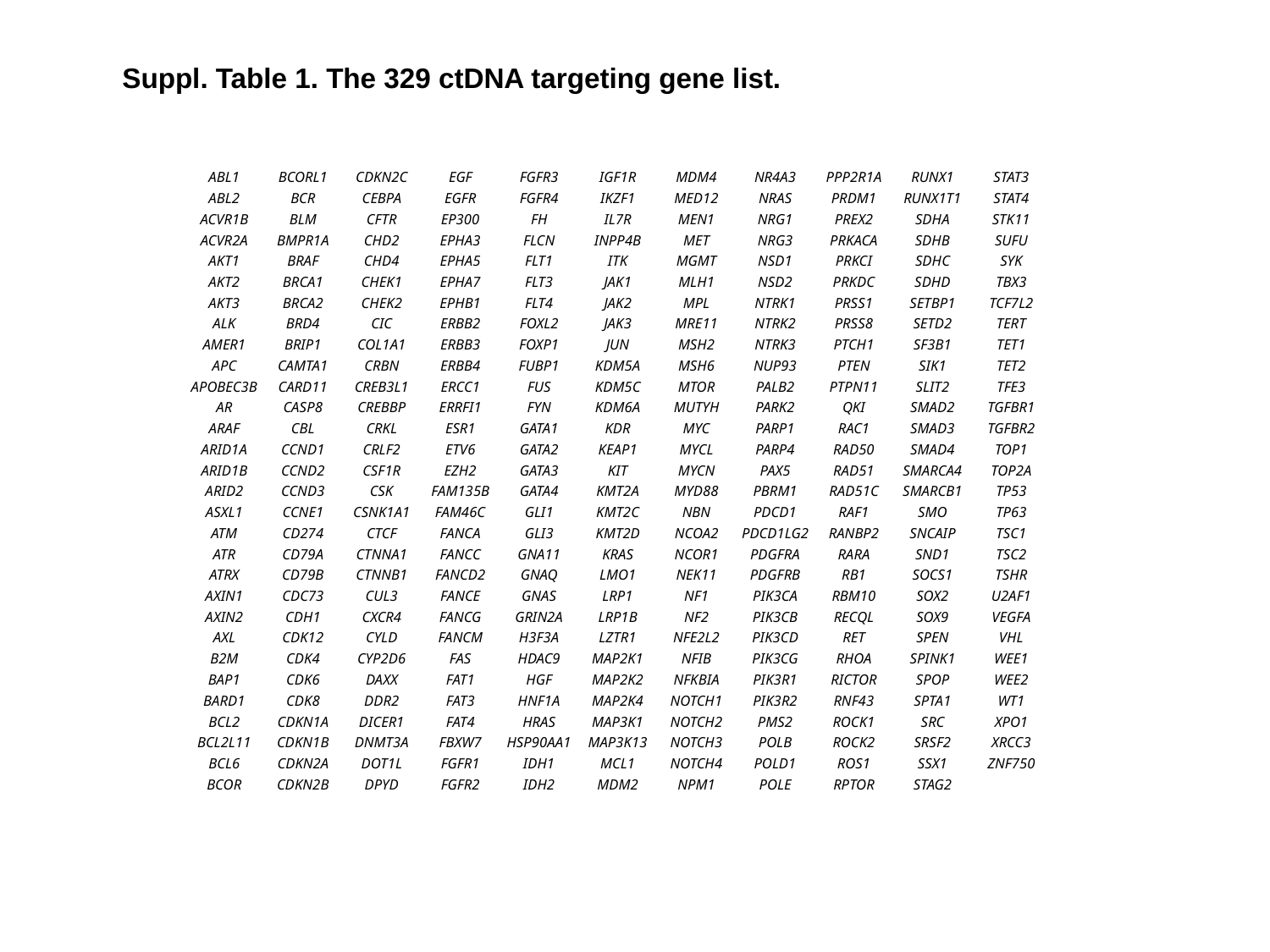

Suppl. Table 1. The 329 ctDNA targeting gene list.
| ABL1 | BCORL1 | CDKN2C | EGF | FGFR3 | IGF1R | MDM4 | NR4A3 | PPP2R1A | RUNX1 | STAT3 |
| --- | --- | --- | --- | --- | --- | --- | --- | --- | --- | --- |
| ABL2 | BCR | CEBPA | EGFR | FGFR4 | IKZF1 | MED12 | NRAS | PRDM1 | RUNX1T1 | STAT4 |
| ACVR1B | BLM | CFTR | EP300 | FH | IL7R | MEN1 | NRG1 | PREX2 | SDHA | STK11 |
| ACVR2A | BMPR1A | CHD2 | EPHA3 | FLCN | INPP4B | MET | NRG3 | PRKACA | SDHB | SUFU |
| AKT1 | BRAF | CHD4 | EPHA5 | FLT1 | ITK | MGMT | NSD1 | PRKCI | SDHC | SYK |
| AKT2 | BRCA1 | CHEK1 | EPHA7 | FLT3 | JAK1 | MLH1 | NSD2 | PRKDC | SDHD | TBX3 |
| AKT3 | BRCA2 | CHEK2 | EPHB1 | FLT4 | JAK2 | MPL | NTRK1 | PRSS1 | SETBP1 | TCF7L2 |
| ALK | BRD4 | CIC | ERBB2 | FOXL2 | JAK3 | MRE11 | NTRK2 | PRSS8 | SETD2 | TERT |
| AMER1 | BRIP1 | COL1A1 | ERBB3 | FOXP1 | JUN | MSH2 | NTRK3 | PTCH1 | SF3B1 | TET1 |
| APC | CAMTA1 | CRBN | ERBB4 | FUBP1 | KDM5A | MSH6 | NUP93 | PTEN | SIK1 | TET2 |
| APOBEC3B | CARD11 | CREB3L1 | ERCC1 | FUS | KDM5C | MTOR | PALB2 | PTPN11 | SLIT2 | TFE3 |
| AR | CASP8 | CREBBP | ERRFI1 | FYN | KDM6A | MUTYH | PARK2 | QKI | SMAD2 | TGFBR1 |
| ARAF | CBL | CRKL | ESR1 | GATA1 | KDR | MYC | PARP1 | RAC1 | SMAD3 | TGFBR2 |
| ARID1A | CCND1 | CRLF2 | ETV6 | GATA2 | KEAP1 | MYCL | PARP4 | RAD50 | SMAD4 | TOP1 |
| ARID1B | CCND2 | CSF1R | EZH2 | GATA3 | KIT | MYCN | PAX5 | RAD51 | SMARCA4 | TOP2A |
| ARID2 | CCND3 | CSK | FAM135B | GATA4 | KMT2A | MYD88 | PBRM1 | RAD51C | SMARCB1 | TP53 |
| ASXL1 | CCNE1 | CSNK1A1 | FAM46C | GLI1 | KMT2C | NBN | PDCD1 | RAF1 | SMO | TP63 |
| ATM | CD274 | CTCF | FANCA | GLI3 | KMT2D | NCOA2 | PDCD1LG2 | RANBP2 | SNCAIP | TSC1 |
| ATR | CD79A | CTNNA1 | FANCC | GNA11 | KRAS | NCOR1 | PDGFRA | RARA | SND1 | TSC2 |
| ATRX | CD79B | CTNNB1 | FANCD2 | GNAQ | LMO1 | NEK11 | PDGFRB | RB1 | SOCS1 | TSHR |
| AXIN1 | CDC73 | CUL3 | FANCE | GNAS | LRP1 | NF1 | PIK3CA | RBM10 | SOX2 | U2AF1 |
| AXIN2 | CDH1 | CXCR4 | FANCG | GRIN2A | LRP1B | NF2 | PIK3CB | RECQL | SOX9 | VEGFA |
| AXL | CDK12 | CYLD | FANCM | H3F3A | LZTR1 | NFE2L2 | PIK3CD | RET | SPEN | VHL |
| B2M | CDK4 | CYP2D6 | FAS | HDAC9 | MAP2K1 | NFIB | PIK3CG | RHOA | SPINK1 | WEE1 |
| BAP1 | CDK6 | DAXX | FAT1 | HGF | MAP2K2 | NFKBIA | PIK3R1 | RICTOR | SPOP | WEE2 |
| BARD1 | CDK8 | DDR2 | FAT3 | HNF1A | MAP2K4 | NOTCH1 | PIK3R2 | RNF43 | SPTA1 | WT1 |
| BCL2 | CDKN1A | DICER1 | FAT4 | HRAS | MAP3K1 | NOTCH2 | PMS2 | ROCK1 | SRC | XPO1 |
| BCL2L11 | CDKN1B | DNMT3A | FBXW7 | HSP90AA1 | MAP3K13 | NOTCH3 | POLB | ROCK2 | SRSF2 | XRCC3 |
| BCL6 | CDKN2A | DOT1L | FGFR1 | IDH1 | MCL1 | NOTCH4 | POLD1 | ROS1 | SSX1 | ZNF750 |
| BCOR | CDKN2B | DPYD | FGFR2 | IDH2 | MDM2 | NPM1 | POLE | RPTOR | STAG2 | |

## Slide 5
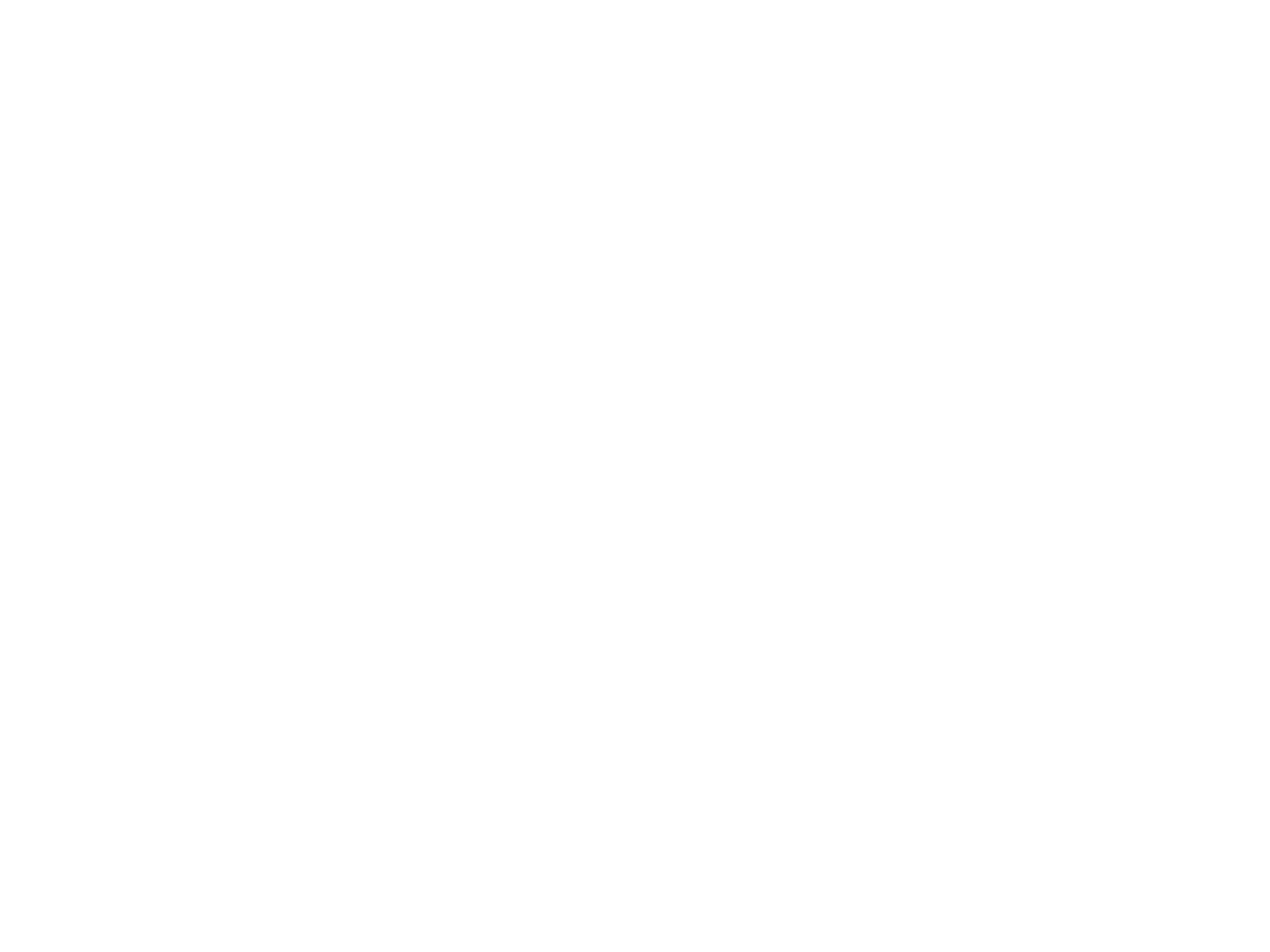

#
