# Supplementary material for: Serial ultra‐deep sequencing of circulating tumor DNA reveals the clonal evolution in non‐small cell lung cancer patients treated with anti‐PD1 immunotherapy
Source: Cancer Med. 2019 Nov 6;8(18):7669–78. doi: 10.1002/cam4.2632 (PMC6912064; doi:10.1002/cam4.2632)
Supplement: Supplementary file 2 [file CAM4-8-7669-s002.docx]

**Suppl. Table 1. The 329 ctDNA targeting gene list.**

| *ABL1* | *BCORL1* | *CDKN2C* | *EGF* | *FGFR3* | *IGF1R* | *MDM4* | *NR4A3* | *PPP2R1A* | *RUNX1* | *STAT3* |
| --- | --- | --- | --- | --- | --- | --- | --- | --- | --- | --- |
| *ABL2* | *BCR* | *CEBPA* | *EGFR* | *FGFR4* | *IKZF1* | *MED12* | *NRAS* | *PRDM1* | *RUNX1T1* | *STAT4* |
| *ACVR1B* | *BLM* | *CFTR* | *EP300* | *FH* | *IL7R* | *MEN1* | *NRG1* | *PREX2* | *SDHA* | *STK11* |
| *ACVR2A* | *BMPR1A* | *CHD2* | *EPHA3* | *FLCN* | *INPP4B* | *MET* | *NRG3* | *PRKACA* | *SDHB* | *SUFU* |
| *AKT1* | *BRAF* | *CHD4* | *EPHA5* | *FLT1* | *ITK* | *MGMT* | *NSD1* | *PRKCI* | *SDHC* | *SYK* |
| *AKT2* | *BRCA1* | *CHEK1* | *EPHA7* | *FLT3* | *JAK1* | *MLH1* | *NSD2* | *PRKDC* | *SDHD* | *TBX3* |
| *AKT3* | *BRCA2* | *CHEK2* | *EPHB1* | *FLT4* | *JAK2* | *MPL* | *NTRK1* | *PRSS1* | *SETBP1* | *TCF7L2* |
| *ALK* | *BRD4* | *CIC* | *ERBB2* | *FOXL2* | *JAK3* | *MRE11* | *NTRK2* | *PRSS8* | *SETD2* | *TERT* |
| *AMER1* | *BRIP1* | *COL1A1* | *ERBB3* | *FOXP1* | *JUN* | *MSH2* | *NTRK3* | *PTCH1* | *SF3B1* | *TET1* |
| *APC* | *CAMTA1* | *CRBN* | *ERBB4* | *FUBP1* | *KDM5A* | *MSH6* | *NUP93* | *PTEN* | *SIK1* | *TET2* |
| *APOBEC3B* | *CARD11* | *CREB3L1* | *ERCC1* | *FUS* | *KDM5C* | *MTOR* | *PALB2* | *PTPN11* | *SLIT2* | *TFE3* |
| *AR* | *CASP8* | *CREBBP* | *ERRFI1* | *FYN* | *KDM6A* | *MUTYH* | *PARK2* | *QKI* | *SMAD2* | *TGFBR1* |
| *ARAF* | *CBL* | *CRKL* | *ESR1* | *GATA1* | *KDR* | *MYC* | *PARP1* | *RAC1* | *SMAD3* | *TGFBR2* |
| *ARID1A* | *CCND1* | *CRLF2* | *ETV6* | *GATA2* | *KEAP1* | *MYCL* | *PARP4* | *RAD50* | *SMAD4* | *TOP1* |
| *ARID1B* | *CCND2* | *CSF1R* | *EZH2* | *GATA3* | *KIT* | *MYCN* | *PAX5* | *RAD51* | *SMARCA4* | *TOP2A* |
| *ARID2* | *CCND3* | *CSK* | *FAM135B* | *GATA4* | *KMT2A* | *MYD88* | *PBRM1* | *RAD51C* | *SMARCB1* | *TP53* |
| *ASXL1* | *CCNE1* | *CSNK1A1* | *FAM46C* | *GLI1* | *KMT2C* | *NBN* | *PDCD1* | *RAF1* | *SMO* | *TP63* |
| *ATM* | *CD274* | *CTCF* | *FANCA* | *GLI3* | *KMT2D* | *NCOA2* | *PDCD1LG2* | *RANBP2* | *SNCAIP* | *TSC1* |
| *ATR* | *CD79A* | *CTNNA1* | *FANCC* | *GNA11* | *KRAS* | *NCOR1* | *PDGFRA* | *RARA* | *SND1* | *TSC2* |
| *ATRX* | *CD79B* | *CTNNB1* | *FANCD2* | *GNAQ* | *LMO1* | *NEK11* | *PDGFRB* | *RB1* | *SOCS1* | *TSHR* |
| *AXIN1* | *CDC73* | *CUL3* | *FANCE* | *GNAS* | *LRP1* | *NF1* | *PIK3CA* | *RBM10* | *SOX2* | *U2AF1* |
| *AXIN2* | *CDH1* | *CXCR4* | *FANCG* | *GRIN2A* | *LRP1B* | *NF2* | *PIK3CB* | *RECQL* | *SOX9* | *VEGFA* |
| *AXL* | *CDK12* | *CYLD* | *FANCM* | *H3F3A* | *LZTR1* | *NFE2L2* | *PIK3CD* | *RET* | *SPEN* | *VHL* |
| *B2M* | *CDK4* | *CYP2D6* | *FAS* | *HDAC9* | *MAP2K1* | *NFIB* | *PIK3CG* | *RHOA* | *SPINK1* | *WEE1* |
| *BAP1* | *CDK6* | *DAXX* | *FAT1* | *HGF* | *MAP2K2* | *NFKBIA* | *PIK3R1* | *RICTOR* | *SPOP* | *WEE2* |
| *BARD1* | *CDK8* | *DDR2* | *FAT3* | *HNF1A* | *MAP2K4* | *NOTCH1* | *PIK3R2* | *RNF43* | *SPTA1* | *WT1* |
| *BCL2* | *CDKN1A* | *DICER1* | *FAT4* | *HRAS* | *MAP3K1* | *NOTCH2* | *PMS2* | *ROCK1* | *SRC* | *XPO1* |
| *BCL2L11* | *CDKN1B* | *DNMT3A* | *FBXW7* | *HSP90AA1* | *MAP3K13* | *NOTCH3* | *POLB* | *ROCK2* | *SRSF2* | *XRCC3* |
| *BCL6* | *CDKN2A* | *DOT1L* | *FGFR1* | *IDH1* | *MCL1* | *NOTCH4* | *POLD1* | *ROS1* | *SSX1* | *ZNF750* |
| *BCOR* | *CDKN2B* | *DPYD* | *FGFR2* | *IDH2* | *MDM2* | *NPM1* | *POLE* | *RPTOR* | *STAG2* |  |
